# Supplementary material for: R2R3-MYB Gene Family in Coptis teeta Wall.: Genome-Wide Identification, Phylogeny, Evolutionary Expansion, and Expression Analyses during Floral Development
Source: Int J Mol Sci. 2024 Aug 15;25(16):8902. doi: 10.3390/ijms25168902 (PMC11354770; doi:10.3390/ijms25168902)
Supplement: Supplementary file 1 [file ijms-25-08902-s001.zip › S-FiguresV3.1.pdf]

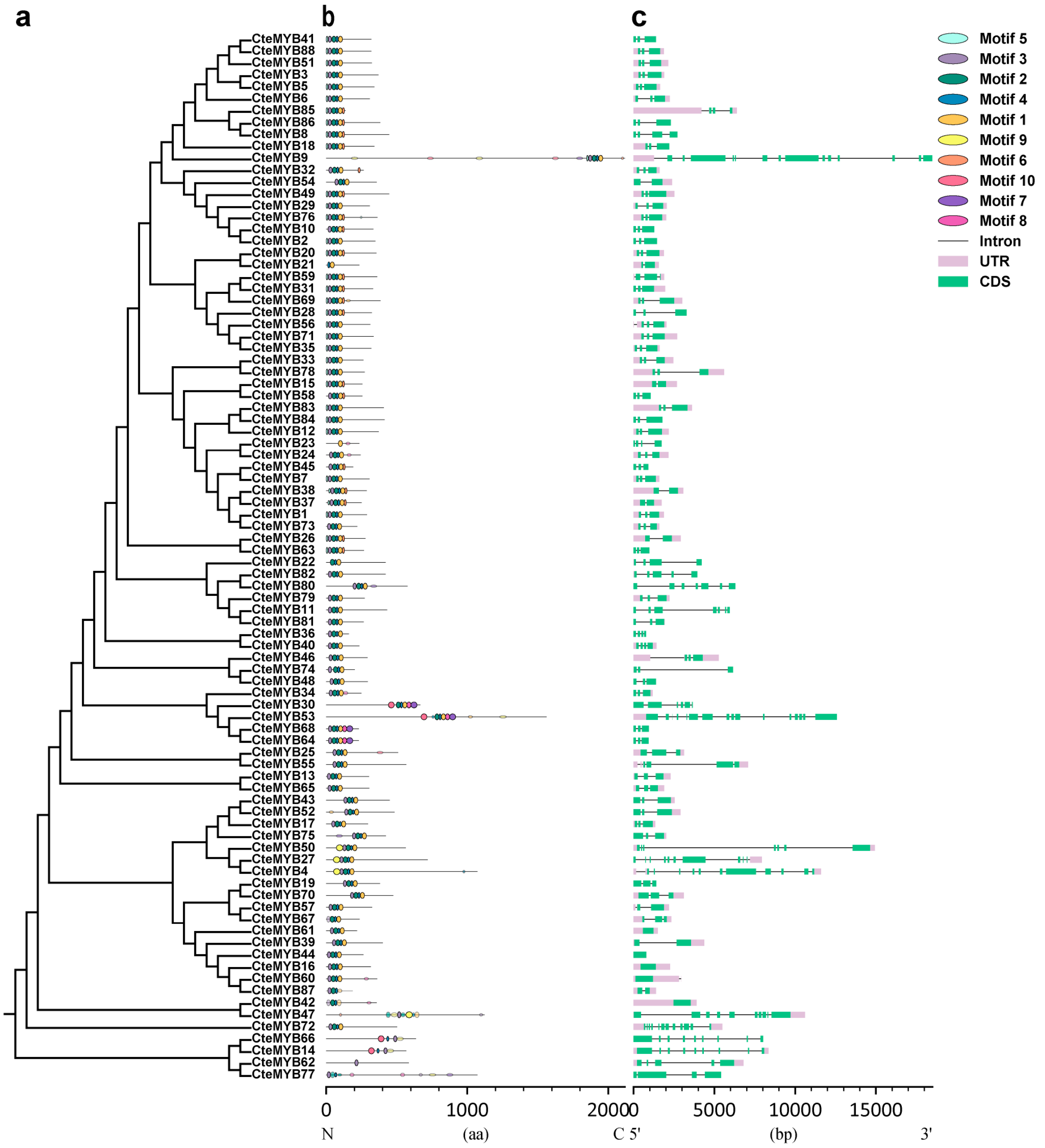

Fig. S1. The phylogenetic tree, conserved motifs, and gene structure structure of *CteR2R3-MYB* genes. a. Phylogenetic tree of *CteR2R3-MYB* genes. b. The distribution of 10 conserved motifs in *CteR2R3-MYB* genes.Distinct conserved motifs are identified through the use of elliptical shapes in various colors. (c) Gene structure of *CteR2R3-MYB* genes. The untranslated region (UTRs), coding sequences (CDS) and introns region

are represented by purple rectangles, green rectangles, grey lines, respectively.

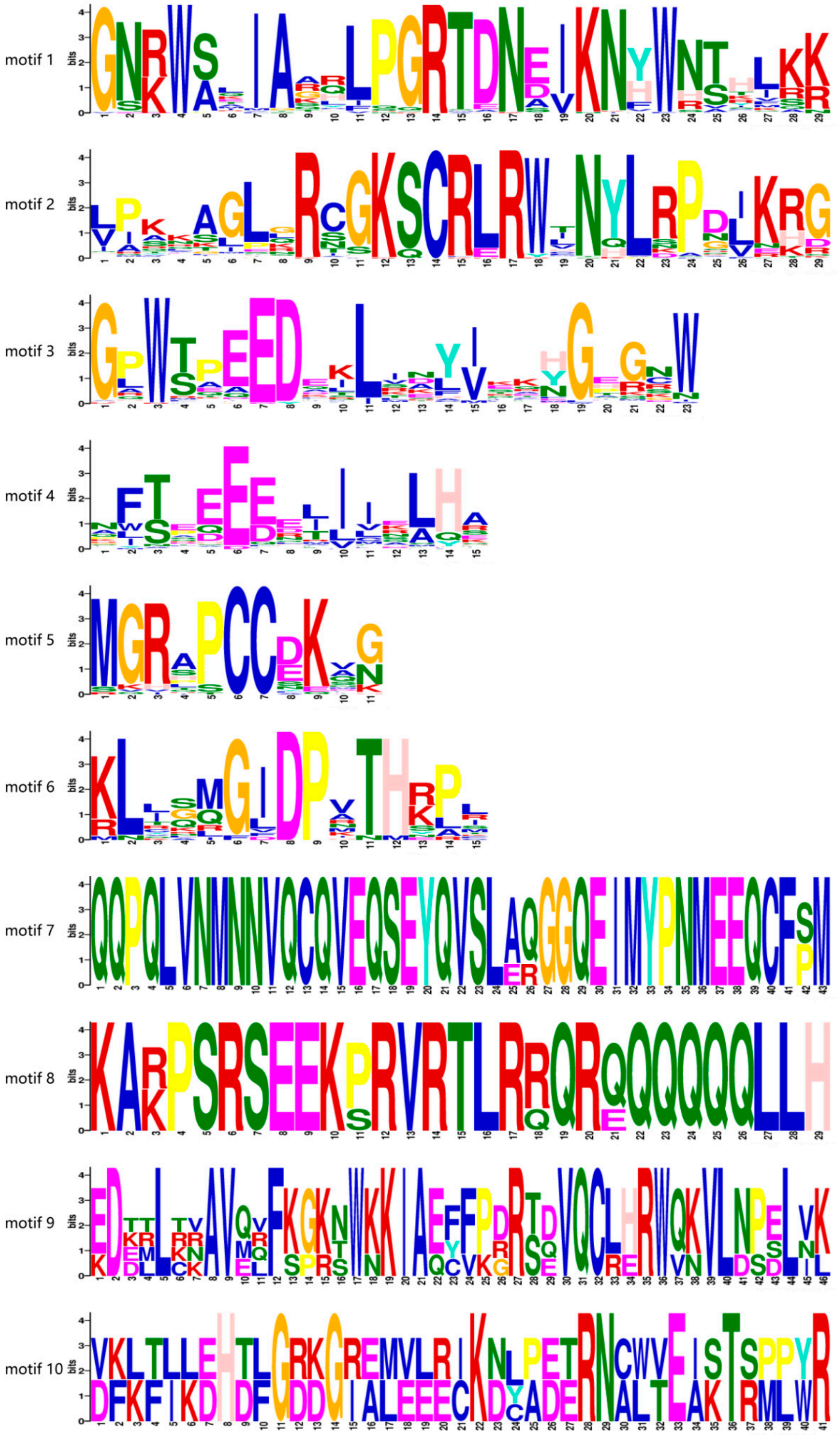

Fig. S2. The sequence logos of 10 conserved motifs in *CteR2R3-MYB*

genes

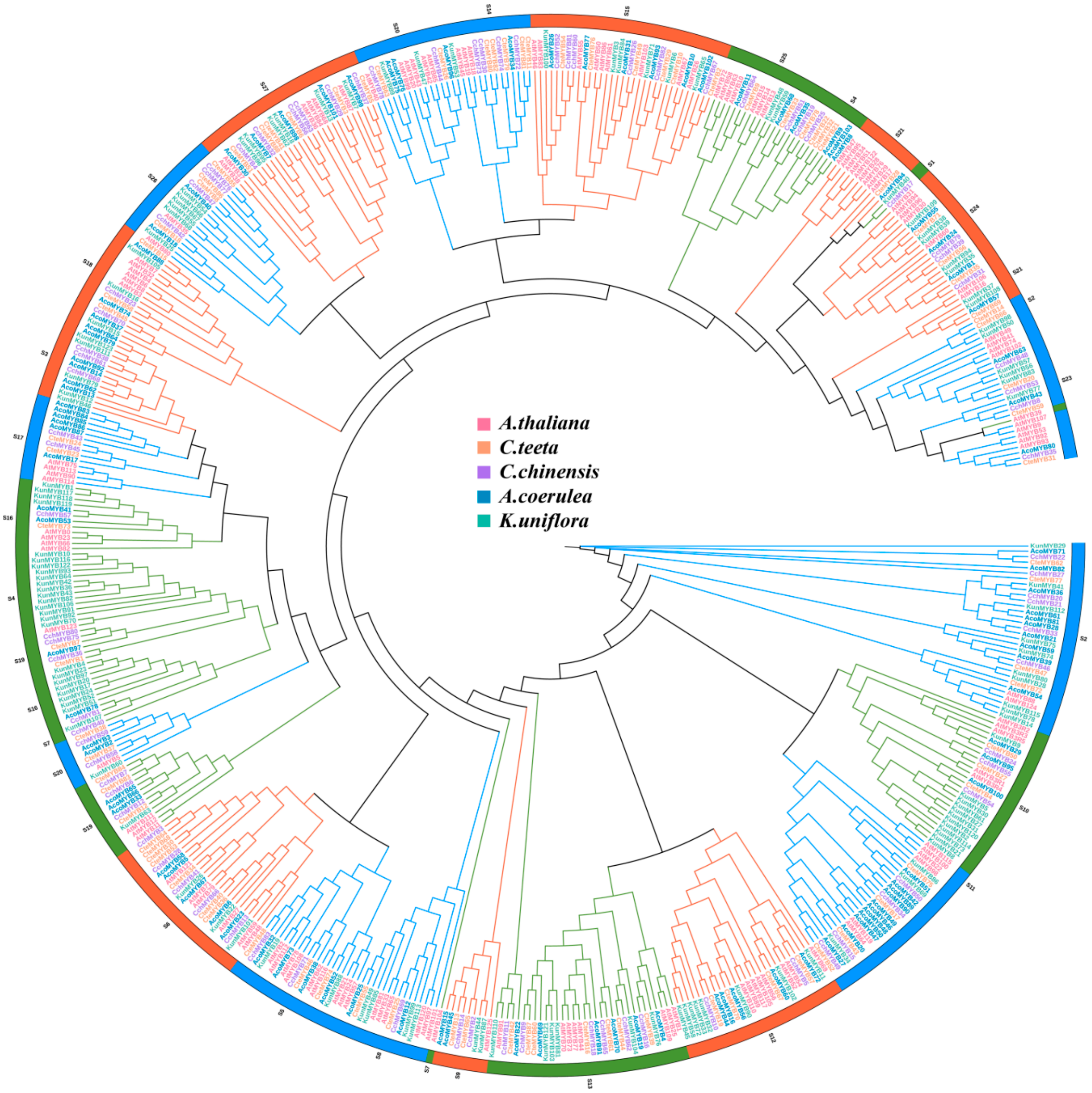

Fig. S3. Phylogenetic relationship of *R2R3-MYBs*. *A.thaliana* is depicted in pink letters, *C.teeta* in yellow, *C.chinensis* in purple, and *A.coerulea* in blue. *K.uniflora* is denoted by green characters. R2R3-MYBs were used for phylogenetic tree construction in five species, using the neighbor-joining method through the MEGA11, and data beautification was performed using iTOL (https://itol.embl.de/).

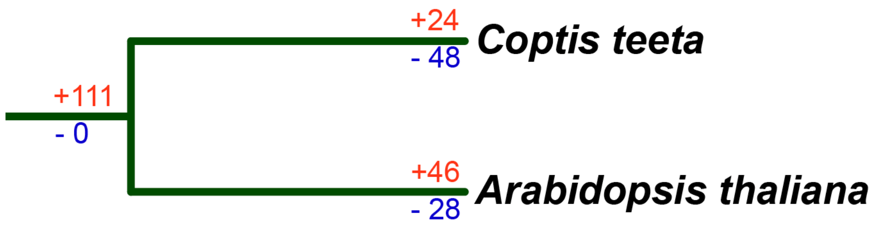

Fig. S4. Gene duplication and loss analysis of R2R3-MYB family genes in both *C.teeta* and *A.thaliana*.Duplication and loss of genes are represented by + and - numbers, respectively.

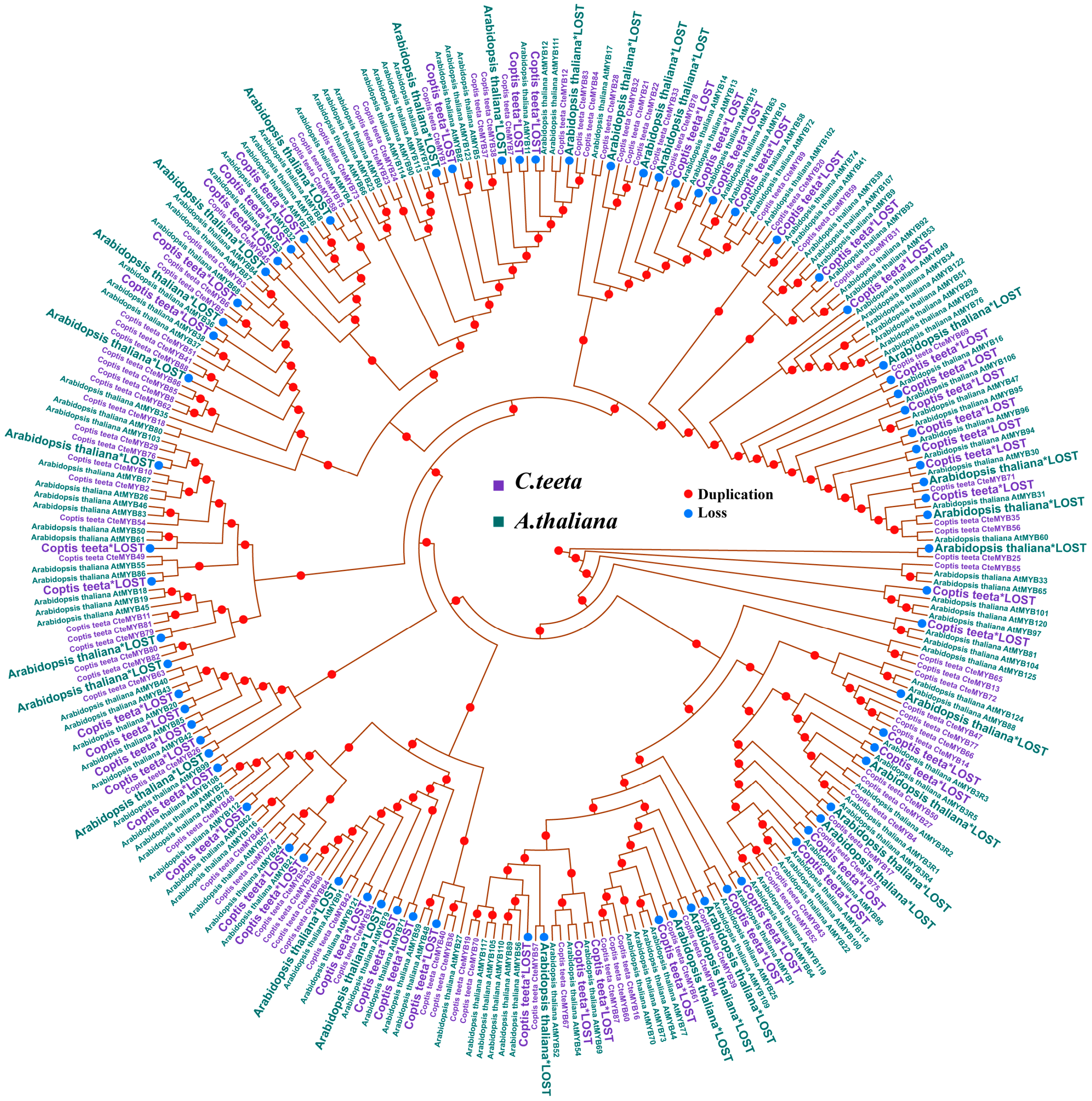

Fig. S5. Proposed evolutionary history of the R2R3-MYB gene family in both *C.teeta* and *A.thaliana*.Duplicate is depicted by red circles, loss is represented by blue circles.

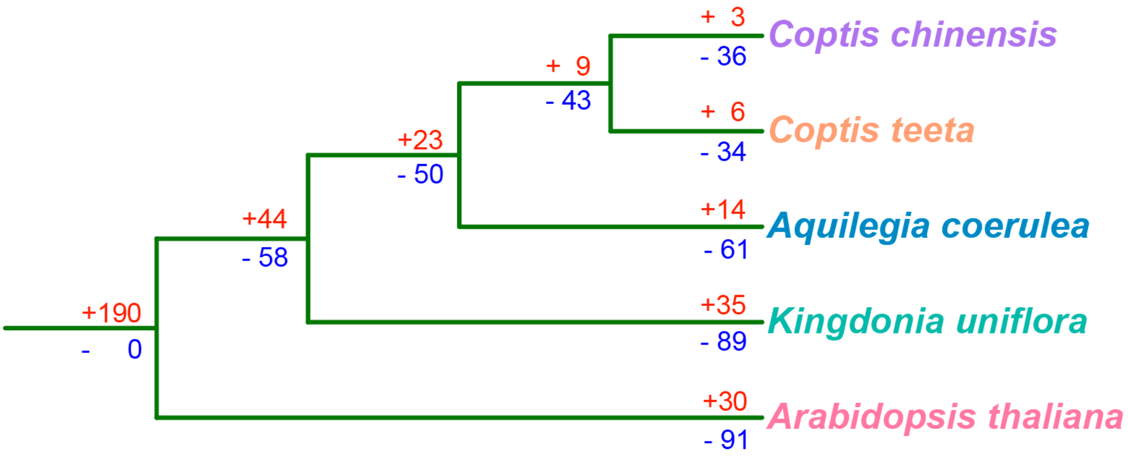

Fig. S6. Gene duplication and loss analysis of R2R3-MYB family genes.Duplication and loss of genes are represented by + and - numbers, respectively.

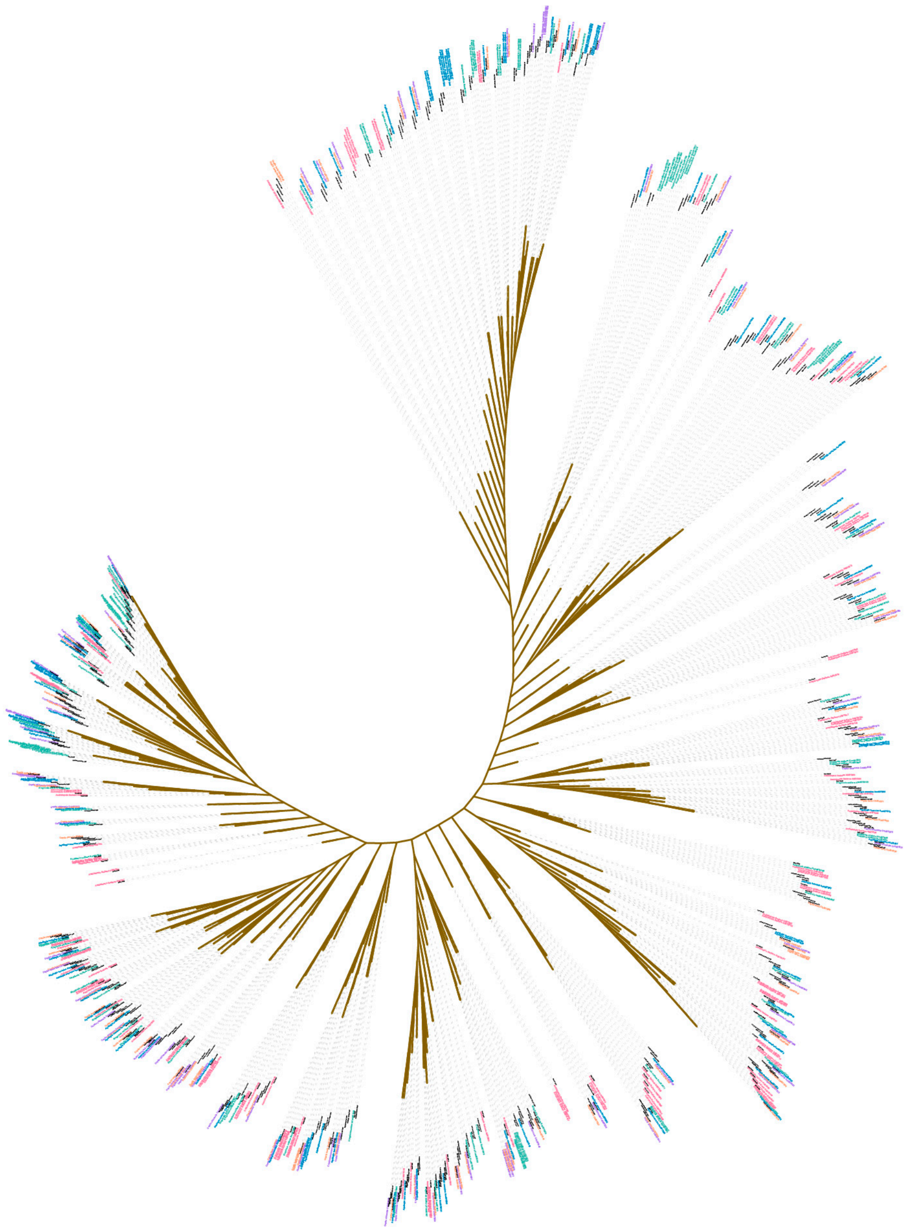

Fig. S7. Proposed evolutionary history of the 527 R2R3-MYB genes. Duplicate is depicted by red letters, loss is represented by blue letters. *A.thaliana* is depicted in pink letters, *C.teeta* in yellow, *C.chinensis* in purple, and *A.coerulea* in blue. *K.uniflora* is denoted by green characters.

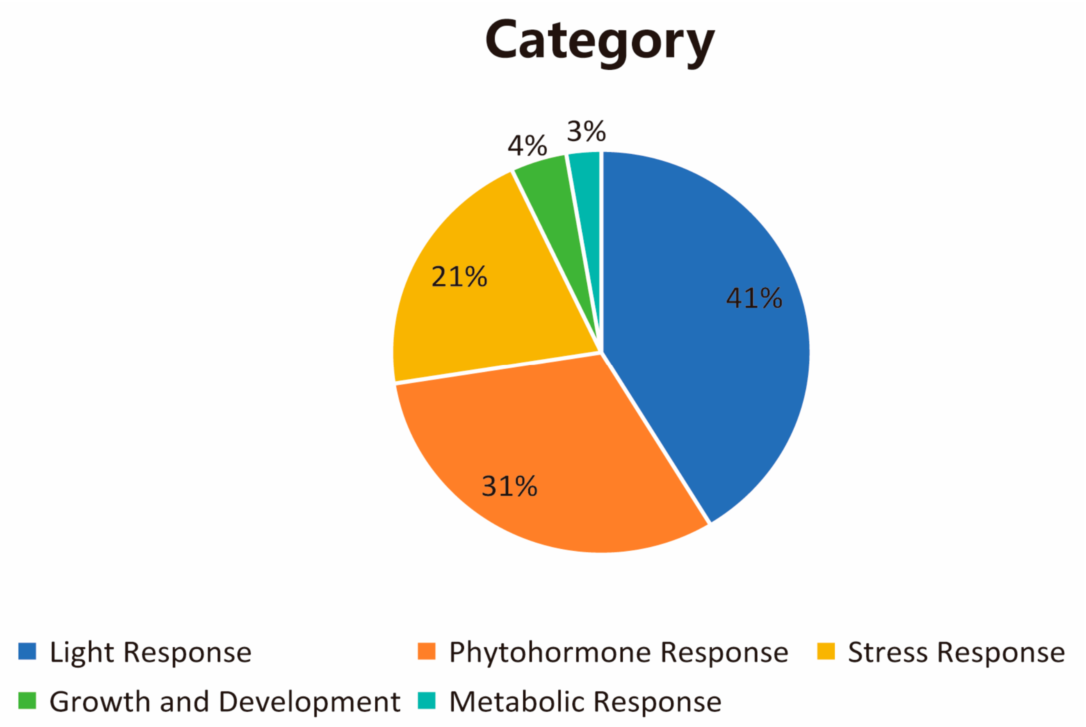

Fig. S8. Percentage-based classification of cis-acting elements within the R2R3-MYB gene in *C.teeta*

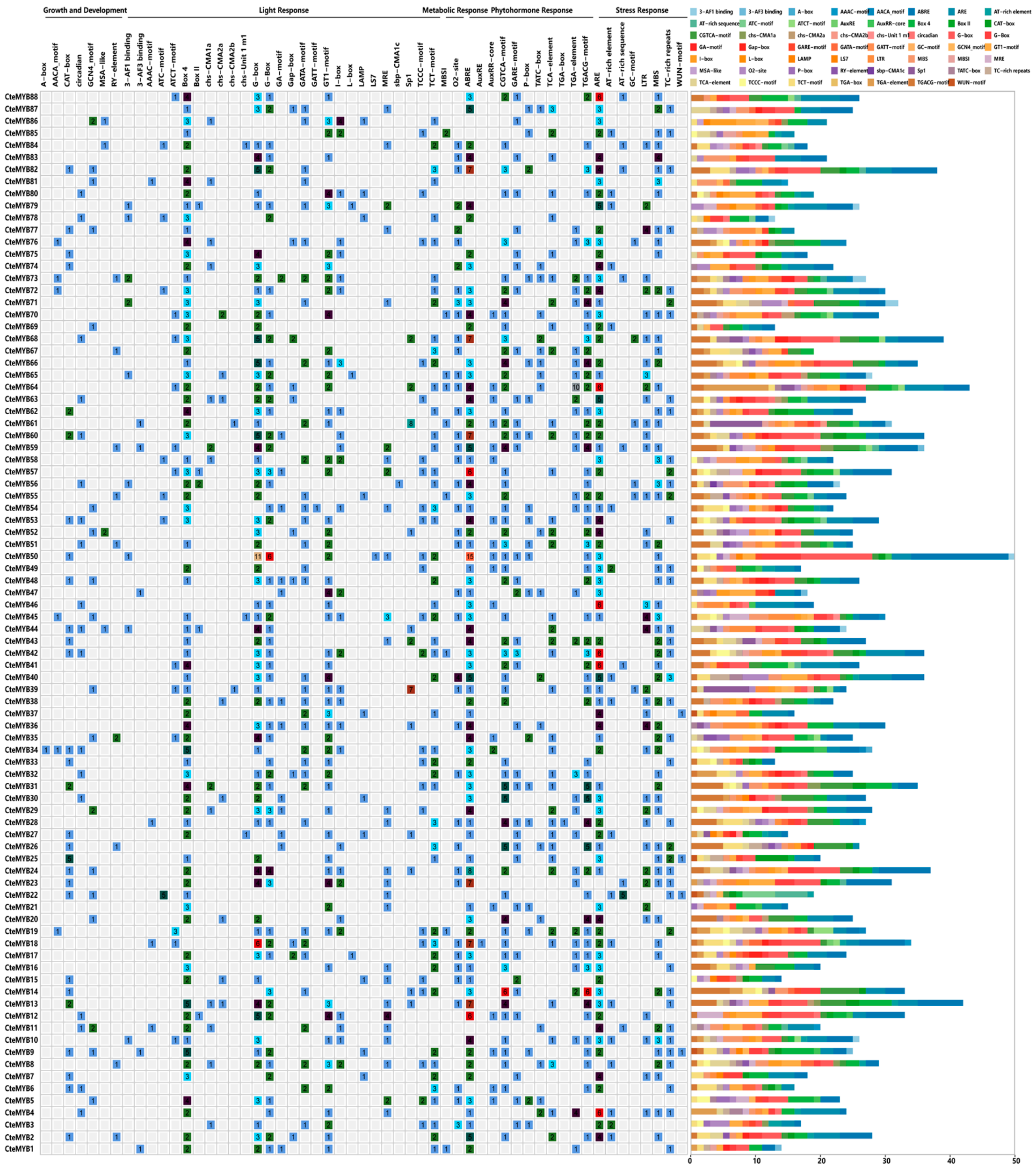

Fig. S9. The promoter region of *CteR2R3-MYB*s includes cis-elements. Left: the number and functional classification of cis-acting elements in each *R2R3-MYB* gene. Right: the distribution of 55 identified cis-acting elements in each *R2R3-MYB* gene is illustrated, with numbers represented by boxes in various colors.

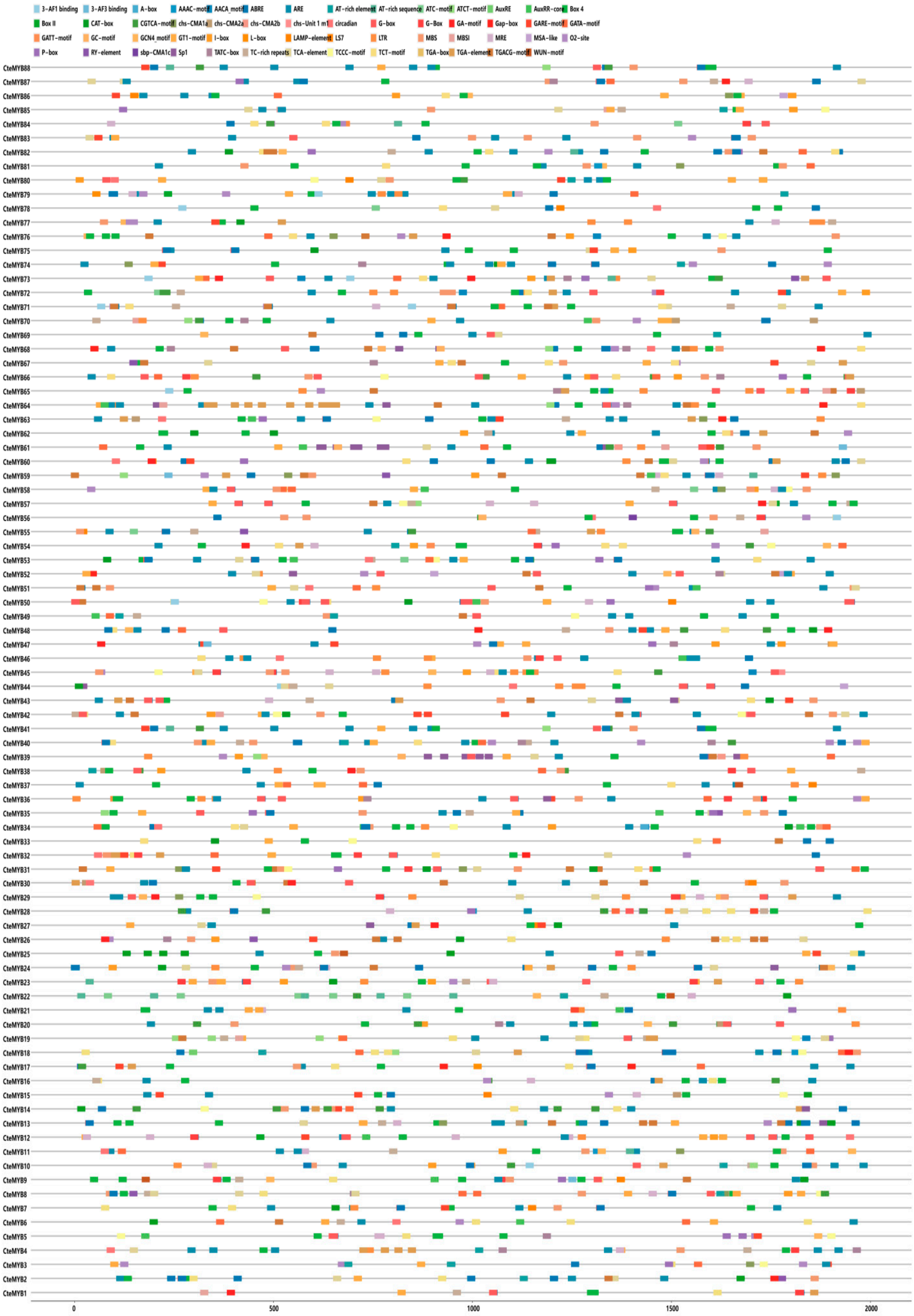

Fig. S10. Visualization of the distribution of cis-elements within the R2R3-MYB genes in *C. teeta*
